# Brachial plexus dose tolerance in head and neck cancer patients treated with sequential intensity modulated radiation therapy

**DOI:** 10.1186/s13014-015-0409-5

**Published:** 2015-04-18

**Authors:** Tarita O Thomas, Tamer Refaat, Mehee Choi, Ian Bacchus, Sean Sachdev, Alfred W Rademaker, Vythialingam Sathiaseelan, Achilles Karagianis, Bharat B Mittal

**Affiliations:** Department of Radiation Oncology, Northwestern University, Robert H. Lurie Comprehensive Cancer Center, 251 East Huron, LC-178, Chicago, IL 60611 USA; Department of Preventive Medicine, Northwestern University, Robert H. Lurie Comprehensive Cancer Center, Chicago, IL USA; Department of Radiology, Northwestern University, Robert H. Lurie Comprehensive Cancer Center, Chicago, IL USA; Northwestern Medicine Developmental Therapeutics Institute (NMDTI), Chicago, IL USA; Department of Clinical Oncology and Nuclear Medicine, Faculty of Medicine, Alexandria University, Alexandria, Egypt

**Keywords:** Brachial plexus, Radiation, Tolerance

## Abstract

**Purpose:**

We aimed to study the radiation induced brachial plexopathy in patients with head and neck squamous cell carcinoma (HNSCC) treated with Sequential Intensity Modulated Radiation Therapy (S-IMRT).

**Methods and materials:**

This IRB approved study included 68 patients with HNSCC treated consecutively. Detailed dose volume histogram data was generated for ipsilateral and contralateral brachial plexus (BP) volumes receiving a specified dose (Vds) i.e. V50-V75 and dose in Gray covering specified percent of BP volume (Dvs) i.e. D5-D30 and maximum point doses (Dmax). To assess BP injury all patients’ charts were reviewed in detail for sign and symptoms of BP damage. Post-hoc comparisons were done using Tukey-Kramer method to account for multiple significance testing.

**Results:**

The mean and maximum doses to BP were significantly different (p < .05) based on tumor site, nodal status and tumor stage. The mean volume to the ipsilateral BP for V50, V60, V70, and V75 were 7.01 cc, 4.37 cc, 1.47 cc and 0.24 cc, respectively. The mean dose delivered to ≤5% of ipsilateral BP was 68.70 Gy (median 69.5Gy). None of the patients had acute or late brachial plexopathy or any other significant neurological complications, with a minimum follow up of two years (mean 54 months).

**Conclusions:**

In this study cohort, at a minimum of two-years follow up, the mean dose of 68.7Gy, a median dose to 69.5Gy to ≤5% of ipsilateral BP, and a median Dmax of 72.96Gy did not result in BP injury when patients were treated with S-IMRT technique. However, longer follow up is needed.

## Summary

Radiation therapy may induce brachial plexopathy. Current Radiation Therapy Oncology Group (RTOG) guidelines suggest dose constraints ranging from 60-66Gy. We found that over a minimum follow-up of two years (mean 54 months) none of our patients developed acute or late brachial plexopathy with a mean, and median dose of 68.7Gy, and 69.5Gy delivered to less than 5% of ipsilateral BP and a median maximum point dose of 72.96 Gy to the ipsilateral BP although longer follow-up is necessary.

## Introduction

Concomitant chemo-radiation (CRT) is the current standard of care for local-regional advanced head and neck squamous cell carcinoma (HNSCC). Intensity Modulated Radiation Therapy (IMRT) is the commonly utilized radiation therapy technique in this setting, either using Sequential IMRT (S-IMRT) [1] or Simultaneous Integrated Boost (SIB-IMRT) techniques [2].

Radiation induced brachial plexopathy is a concerning adverse event among HNSCC patients; it is defined as neurologic impairment of a transient or permanent nature as a sequela to radiation therapy [3]. Symptoms include paresthesia, pain, weakness, and motor dysfunction affecting the chest, shoulder and upper extremity [4]. The Radiation Therapy Oncology Group (RTOG) has endorsed an atlas that has been developed and validated for delineating the brachial plexus (BP), which has helped in standardizing BP contouring [5-7]. Limits on the dose to the BP in patients receiving IMRT have been recommended by the RTOG to 60–66 Gy on most RTOG clinical protocols including; RTOG 0435 (≤60 Gy), RTOG 0522 (≤60 Gy), RTOG 0412 (Dmax 60 Gy), and RTOG 0615 (≤66 Gy). However, based on tumor location and stage, some patients may be treated to higher doses of radiation to the BP. Furthermore, radiation therapy induced brachial plexopathy may vary depending on dose per fraction, total dose and volume of BP exposed to radiation and the use of CRT [3]. Most of the published data regarding dose to the BP are based on 2D/3D or SIB-IMRT planning techniques. This is the first report evaluating the dose/volume relationship to radiation-induced brachial plexopathy in patients with locally advanced HNSCC treated with S-IMRT.

## Methods

### Patient selection & adverse event reporting

The institutional review board of Northwestern University approved this study. From January 2003 to December 2008 a total of 68 patients with HNSCC were treated with S-IMRT. Patients were treated to a prescribed dose of 54–72 Gy with or without concurrent chemotherapy. Lower doses were prescribed to patients treated in adjuvant fashion. All patients had a minimum of two years follow-up. Brachial plexopathy was retrospectively determined according to the Common Terminology Criteria for Adverse Events version 3.0.

### Immobilization and simulation

Patients were immobilized using an Aquaplast face mask (WFR/Aquaplast Corp, Wyckoff, New Jersey). Treatment planning CT scan was performed using IV contrast in the majority of patients. CT imaging included the region between the vertex and carina with 3 mm slice thickness.

### Target volumes definition

The details of S-IMRT target volume definition and treatment planning have been reported [1]. The following guidelines were used:

1. *Definitive S-IMRT:*

The gross tumor volume (GTV) included clinically and/or radiologically visible disease. Clinical target volume 1 (CTV_1_) included GTV and high and low risks elective nodal regions in the neck with a 1 – 2 cm margin. Clinical target volume 2 (CTV_2_) included GTV and high-risk elective nodal regions with 0.75 – 1.5 cm margin. Clinical target volume 3 (CTV_3_) included GTV expanded by 0.5 – 1 cm to cover any microscopic soft tissue extension.

Three planning target volumes (PTV 1 – 3) were created that encompassed the corresponding CTV with a margin of 3 – 5 mm to account for set up and patient movement errors. Later on, for sake of simplicity, we only outlined GTV and three PTVs that encompassed corresponding CTVs as defined above. We used axial images from the planning CT to identify nodal levels in the neck as described by Som et al. [8]. We largely used the historical data for neck metastases summarized by Chao et al. [9] to stratify nodal disease as high-risk and low-risk for elective neck irradiation.

2. *Post-operative S-IMRT:*

The surgical bed was defined as the area of pre-operative GTV and the operated area that included the resected tumor, involved lymph nodes and post-surgical changes. CTV_1_ included the surgical bed and regional high and low risk elective nodal chain with 1–2 cm margin. CTV_2_ included surgical bed and high-risk elective nodal chain with 1 – 2 cm margin. In patients with high-risk features like extra-capsular extension (ECE) the surrounding soft tissue was included with a generous margin. Similar to definitive S-IMRT, each CTV was expanded by 3 – 5 mm to create PTV_1_ and PTV_2_ and later on for simplicity, the surgical bed and 2–3 PTVs were created. In both definitive as well as post-operative cases, the S-IMRT target volumes were drawn 2 – 3 mm deeper to the skin to spare dermal structures unless the gross tumor or ECE was very close to the skin.

### Dose specifications

Radiation doses were sequentially prescribed using conventional fractionation of 180 – 200 cGy to all patients. This study included only patients who received once a day, five-days a week, conventional fractionation and excluded patients who were treated using hyperfractionated or accelerated fractionation schemes. Typically, the low risk target volume (PTV_1_) was treated to 45 – 50 Gy, the intermediate to high-risk target volume (PTV_2_) was treated to 56 – 66 Gy and the gross tumor with expansion (PTV_3_) was treated to 66 – 72 Gy. Three separate IMRT plans corresponding to each PTV were generated and whole neck IMRT was used.

### Dose optimization

Treatment planning utilized 7 – 9 beams inverse planning IMRT technique. For all target PTVs, maximum dose was used to restrict dose to <110% of the prescribed dose, whereas maximum dose-volume histograms (DVH) was used to restrict the 105% dose volume inside PTV and minimum DVH used to ascertain that at least 95% of the PTV is covered by 100% of the prescribed dose.

For normal structures, a combination of max dose, max DVH, and max Equivalent Uniform Dose were used as optimization criteria. To achieve a clinically acceptable plan, the objective values and weights were iteratively adjusted with the following order of priority: (1) minimize the maximum dose to the serial organs such as the spinal cord and brainstem; (2) minimize the mean dose to the swallowing organs at risk (OARs) such as the pharyngeal constrictors, larynx, postcricoid esophagus, as well as BP; and (3) minimize the mean dose to parotids and oral cavity.

### Computational method for analysis of dose to BP

For each patient, the Pinnacle Treatment Planning system was used to generate a text file that contained DVH data, PTV volumes, and treatment plan statistics. MATLAB (version 2012a) was then used to format and parse the data into an Excel workbook for further analysis. The total prescribed dose was calculated by fraction size and the number of individual prescriptions by assigning a dose in cGy to the volumes in the DVH based on the bin size and number (i.e., bin 1 would be 0 cGy, bin 2 would be 50 cGy, bin 3 would be 100 cGy, etc.). The percent of total prescribed dose to each total region of interest (ROI) volume was then calculated. In order to verify that values calculated by the MATLAB programs were correct, calculations were replicated in Excel with the raw data. The calculated values from MATLAB agreed with the Excel-generated values to within an average of 0.003%.

### DVH analysis of BP dose

The volume of BP receiving a specified dose in Grays (Vds) was computed in cc (i.e. V50 – V75). The dose in Gray covering a specified percent of BP volume (Dvs) was also tabulated (i.e. D5 – D30). The mean, median and maximum, V50, V60, V70, V75, D5, D10, D15, D20, D25, D30 values of ipsilateral and contralateral BP were calculated based on tumor site and stage.

### Brachial plexus contouring technique

**Figure 1:**
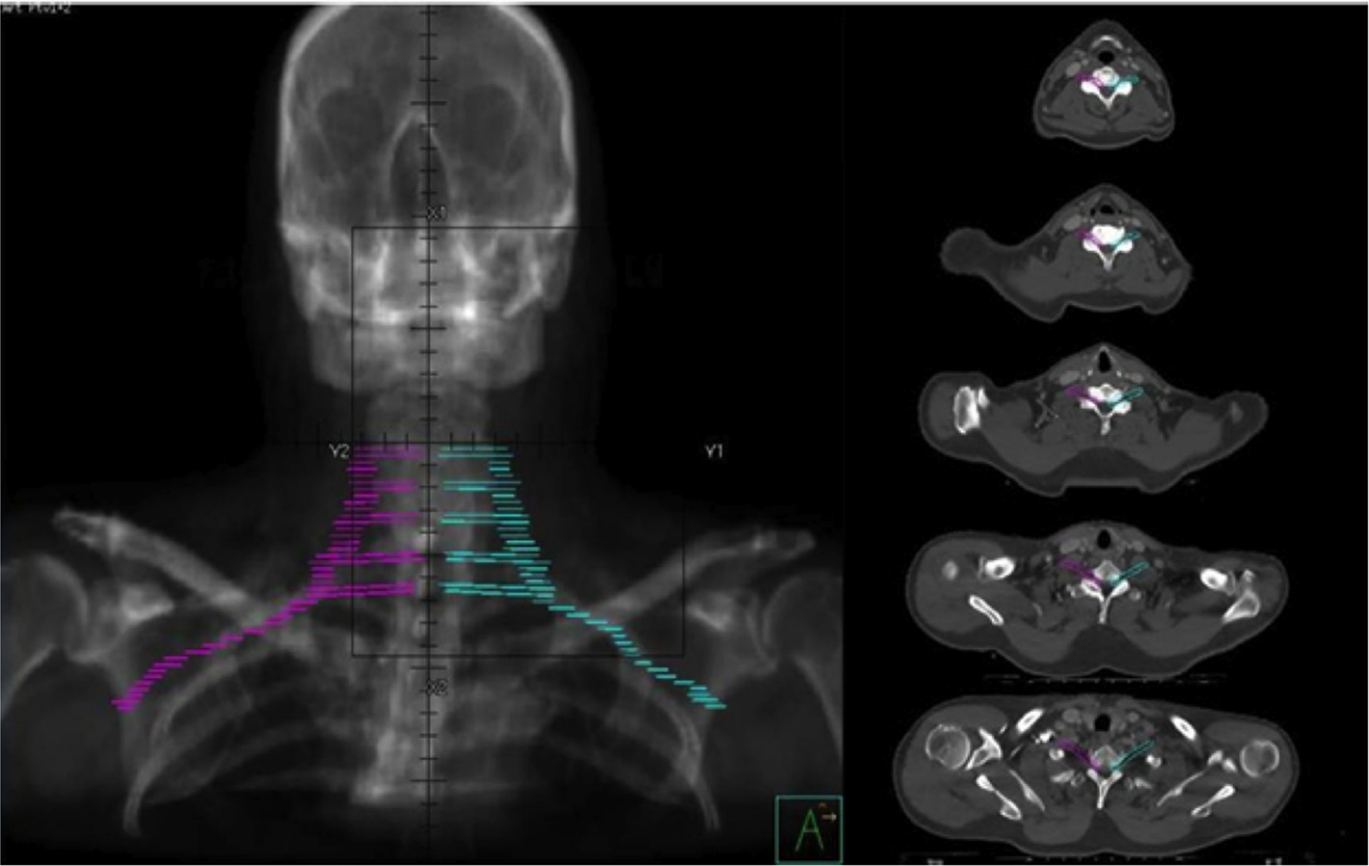
Digital reconstructed radiograph of contoured brachial plexus with the right BP in magenta and left BP in cyan. Right panel shows axial images of representative slices contoured with the right and left BP.

The BP was contoured on both sides per the RTOG-endorsed brachial plexus contouring atlas [5]. The right and left brachial plexi were contoured with a 3-5-mm diameter paint tool as separate regions of interest (ROI) in all patients. The ROIs were delineated by one radiation oncologist and reviewed and adjusted when considered appropriate by a second radiation oncologist. Figure 1 shows an example of our BP-contouring technique.

### Follow up

Patients were seen in follow-up at 1 month after completion of radiation, followed by approximately every 3 months in the first year, every 4 months in the 2nd year and every 6 months for five years then with yearly follow-ups thereafter. The first imaging study CT and or PET scan was obtained 1–3 months after treatment to assess response and thereafter as indicated. Patients usually had an imaging study performed once a year. Radiation induced brachial plexopathy was diagnosed if the affected side was within the prior radiation field and the symptoms were not due to another known etiology (i.e. tumor progression or acute injury). All adverse events were retrospectively reported based on the Common Terminology Criteria for Adverse Events version 3.0 (CTCAE v3.0). Based on CTCAE v3.0 injury to the BP was graded 1–5 as: grade 1 was asymptomatic, grade 2 was symptomatic not interfering with activities of daily living, grade 3 was symptomatic and interfering with activities of daily living, grade 4 was disabling and grade 5 was death. As per our institutional practice, regular follow up for HNSCC patients included evaluation of treatment-induced adverse events that were graded according to CTCAE v3.0 and reported in the patients’ charts.

### Statistical analysis

Dose parameters were compared across categories of clinical variables (tumor site, nodal status, stage) using analysis of variance, accounting for different levels of variation across categories. Post-hoc comparisons were done using the Tukey-Kramer method, which accounts for multiple significance testing [10]. Statistical significance was indicated when p < 0.05.

## Results

### Patient and tumor demographics

Table 1 lists patient and tumor characteristics for the 68 patients in the study. Notably, most patients were male (84%) and most had stage IV disease (76.47%). Thirty-eight percent of patients had T2 disease and 61.8% of patients had N2 disease. The oropharynx (48.5%) was the most commonly treated site. Fifty patients were treated definitively with the remainder treated adjuvantly and 86.8% of cases were treated with chemotherapy.

**Table 1:** **Patient and tumor characteristics**

**Characteristics**			
**Age (in years)**	Mean	56	
	Median	55	
	Range	30-89	
**Follow up (in months)**	Mean	54	
	Median	49	
	Range	24-108	
		**N**	**%**
**Sex**	Male	57	84%
	Female	11	16%
	Total	68	100%
**Group stage**	I*	1	1.5%
	II	3	4.4%
	III	8	11.8%
	IV	52	76.47%
	Total	68	100%
**T status**	T0	10	14.7%
	T1	11	16.2%
	T2	26	38.2%
	T3	10	14.7%
	T4	11	16.2%
	Total	68	100%
**N stage**	N0	13	19.1%
	N1	8	11.8%
	N2	42	61.8%
	N3	5	7.4%
	Total	68	100%
**Primary tumor site**	Oral Cavity	6	8.8%
	Oropharynx	33	48.5%
	Nasopharynx	9	13.2%
	Hypopharynx	3	4.4%
	Larynx	7	10.3%
	Unknown Primary	10	14.7%
	Total	68	100%
**Aim of treatment**	Definitive	50	73.5%
	Adjuvant + Postop	18	26.5%
	Total	68	100%
**Chemotherapy**	Yes	59	86.8%
	No	9	13.2%
	Total	68	100%

*Nasopharynx Squamous Cell Carcinoma.

### Tumor site, stage and BP dose association

The mean volume of BP was 11.6 cc (Median, 11.6 cc). Table 2 shows the association between components of the DVH for both ipsilateral and contralateral BP compared across categories of clinical variables including tumor site, nodal status, and tumor group stage. For ipsilateral BP, comparisons of DVH components across sites were significant except for volume of BP receiving >70Gy (V70) or >75Gy (V75). When significant, these components were lowest for oral cavity and hypopharynx tumors. Comparisons of ipsilateral DVH components across nodal status were significant for all components with N0 and N1 tumors having lower values than N2 and N3 nodes. Comparisons of ipsilateral tumors by stage of disease were significant only for V60, D10, and D20 where stage III or lower stage tumors had lower values than stage IV tumors. DVH components for the contralateral BP were significant across sites except for volume of BP receiving >70Gy (V70) and >75Gy (V75). When significant, oral cavity and oropharyngeal tumors had lower values than other tumors. Based on nodal status, contralateral DVH components were significant except for the maximum dose, volume of BP receiving >70Gy (V70) and >75Gy (V75), and when significant, showed an increase in values with increasing nodal stage. However when stage group was evaluated for dose parameters for the contralateral BP only mean dose, and volume of BP receiving >50Gy were significantly different with lower stage tumors having lower values.

**Table 2:** **Association between tumor site, nodal status, group stage and BP dose**

**Combined BP (n = 136)**	**SITE (OC, OPhx, NPhx, Larynx, UNP, HPhx)**	**N status (N0 – N3)**	**Group stage (I-III vs. IV)**
Mean dose	0.005	0.0002	0.037
Max dose	0.0002	0.002	0.17
V50cc	0.012	< .0001	0.049
V60cc	0.0003	< .0001	0.08
V70cc	0.002	< .0001	0.33
V75cc	0.19	0.08	0.039
D5	0.0005	0.0001	0.12
D10	< .0001	< .0001	0.026
D15	0.0006	< .0001	0.06
D20	0.003	< .0001	0.022
D25	0.22	< .0001	0.10
D30	0.002	< .0001	0.07
**Ipsilateral BP (n = 68)**	**SITE**	**N status (N0 – N3)**	**Group stage (I-III vs. IV)**
Mean dose	< .0001	< .0001	0.06
Max dose	0.038	0.021	0.20
V50cc	0.004	0.001	0.10
V60cc	0.019	< .0001	0.010
V70cc	0.53	< .0001	0.06
V75cc	0.53	0.001	0.28
D5	0.004	0.0004	0.11
D10	0.003	< .0001	0.007
D15	0.001	0.0006	0.08
D20	0.001	< .0001	0.021
D25	0.013	0.0003	0.14
D30	0.0004	0.0004	0.06
**Contralateral BP (n = 68)**	**SITE (OC, OPhx, NPhx, Larynx, UNP, HPhx)**	**N status (N0 – N3)**	**Group stage (I-III vs. IV)**
Mean dose	< .0001	0.004	0.045
Max dose	< .0001	0.10	0.29
V50cc	0.003	0.006	0.041
V60cc	< .0001	0.021	0.44
V70cc	0.15	0.20	0.66
V75cc	0.15	0.86	0.52
D5	< .0001	0.042	0.21
D10	< .0001	0.030	0.14
D15	0.0001	0.004	0.09
D20	< .0001	0.009	0.11
D25	< .0001	0.018	0.19
D30	< .0001	0.006	0.13

OC = oral cavity, OPhx = oropharynx, NPhx = nasopharynx, UNP = unknown primary, HPhx = Hypopharynx.

### Volume of brachial plexus receiving dose (cc)

For the ipsilateral BP the mean volume receiving more than 60Gy (V60) was 4.37 cc or 37.74% (median 3.92 cc or 36%) and 70Gy (V70) was 1.47 cc or 12.74% (median 44 cc or 3.84%). Whereas the mean volume to the contralateral BP receiving more than 60Gy (V60) was 2.0 cc or 17.9% (median 0.5 cc or 4.56%) and 70Gy (V70) was 0.43 cc or 3.74% (median 0 cc). Of note the median V70Gy for the ipsilateral BP was 3.84% of the BP volume (0.44 cc), implying that at least 50% of the patients had a <5% of the ipsilateral BP volume receiving ≥ 70 Gy (Table 3).

**Table 3:** **Volume of BP receiving 50 Gy, 60 Gy, 70 Gy, and 75 Gy**

**Combined BP (n = 136)**	**V50Gy**		**V60Gy**		**V70Gy**		**V75Gy**	
	**cc**	**%**	**cc**	**%**	**cc**	**%**	**cc**	**%**
Mean	6.27	54.11	3.18	27.82	0.95	8.24	0.13	1.13
Median	6.62	62.60	2.47	23.20	0.00	0.00	0.00	0.00
Maximum	14.93	89.20	12.35	86.00	8.06	75.80	6.61	62.18
**Ipsilateral BP (n = 68)**	**V50Gy**		**V60Gy**		**V70Gy**		**V75Gy**	
	**cc**	**%**	**cc**	**%**	**cc**	**%**	**cc**	**%**
Mean	7.01	60.23	4.37	37.74	1.47	12.74	0.24	2.01
Median	7.46	66.70	3.92	36.00	0.44	3.84	0.00	0.00
Maximum	14.93	89.20	12.35	86.00	8.06	75.80	6.61	62.18
**Contralateral BP (n = 68)**	**V50Gy**		**V60Gy**		**V70Gy**		**V75Gy**	
	**cc**	**%**	**cc**	**%**	**cc**	**%**	**cc**	**%**
Mean	5.53	48.00	2.00	17.90	0.43	3.74	0.03	0.26
Median	5.49	49.10	0.50	4.56	0.00	0.00	0.00	0.00
Maximum	12.06	87.80	11.13	77.60	7.51	54.70	1.20	11.71

The volumes of BP in centimeters cubed (cc) and in percent volume (%) are calculated for the mean, median and maximum doses to the combined, ipsilateral and contralateral BP.

### Percentage of brachial plexus receiving dose (%)

For ipsilateral BP (n = 68) the mean dose delivered to ≤ 5% of the BP volume (D5) was 68.7 Gy (median 69.5 Gy, range 45 – 79.25 Gy), to 10% (D10) was 67.37Gy (median 68.50Gy, range 35.5 – 78 Gy) and to 15% (D15) was 64.98 Gy (median 61Gy, range 10.5 – 77.25 Gy). The mean and median maximum point doses (Dmax) to the ipsilateral BP were 71.31 Gy, and 72.96 Gy respectively (range: 48.99 Gy – 78.17 Gy). The mean, median, and maximum D5%, 10%, 15%, 20%, 25%, 30%, as well as Dmax were also calculated for the ipsilateral and contralateral BP (Table 4). Of note the mean D5% to the contralateral BP was 59.07Gy (median 60Gy, range 15Gy - 75Gy), and the median Dmax to the contralateral BP was 62.24 Gy (mean 61.77 Gy, range 18.79 Gy – 76.16 Gy).

**Table 4:** **Percent of brachial plexus receiving given dose in Gray (Gy)**

**Combined BP (n = 136)**	**Dmax (Gy)**	**D5% (Gy)**	**D10% (Gy)**	**D15% (Gy)**	**D20% (Gy)**	**D25% (Gy)**	**D30% (Gy)**
Mean	66.54	63.85	62.46	60.17	59.48	58.23	56.91
Median	68.73	65.50	63.50	61.00	61.80	59.50	58.00
Maximum	78.74	78	77.5	77	76.50	76.00	75.5
**Ipsilateral BP (n = 68)**	**Dmax (Gy)**	**D5% (Gy)**	**D10% (Gy)**	**D15% (Gy)**	**D20% (Gy)**	**D25% (Gy)**	**D30% (Gy)**
Mean	71.31	68.70	67.37	64.98	63.53	62.52	60.90
Median	72.96	69.50	68.50	66.25	63.50	63.50	62.25
Maximum	78.74	78	77.5	77	76.50	76.00	75.5
**Contralateral BP (n = 68)**	**Dmax (Gy)**	**D5% (Gy)**	**D10% (Gy)**	**D15% (Gy)**	**D20% (Gy)**	**D25% (Gy)**	**D30% (Gy)**
Mean	61.77	59.07	57.78	55.60	55.03	53.87	53.04
Median	62.236	60.00	59.75	57.50	57.00	55.00	54.00
Maximum	76.16	75.00	74.40	74.00	73.90	73.50	73.00

The dose delivered to a specified percent (5% or D5%, 10% or D10%, 15% or D15%, 20% or D20%, 25% or D25%, 30% or D30%) of BP in Gy was calculated for the mean, median and maximum doses to the combined, ipsilateral and contralateral BP.

### Follow up & toxicity

The minimum follow up was 2 years (mean 54 months; range 24–108 months) for our patient cohort. A substantial percentage of our patients received higher than recommended doses to BP due to advanced tumor stage or location without adverse events. Three patients developed BP symptoms. However no case was due to radiotherapy or disease progression. One patient acutely developed grade 2 weakness in the ulnar nerve distribution after being hospitalized for systemic infection. MRI in this case revealed diskitis from vertebral levels C7-T1 with evidence of a phlegmon encompassing the associated nerve roots. Another patient acutely developed grade 3 right hand weakness immediately after a traumatic motorcycle injury, with imaging revealing fracture of vertebral levels C6-C7 and significant soft tissue injury. A third patient developed grade 1 finger parasthesias with workup imaging revealing degenerative disk disease with severe cervical spinal stenosis as well as neuroformainal stenosis, corresponding to the affected dermatome.

## Discussion

In spite of high dose none of the patients experienced signs or symptoms of radiation induced BP injury; we are presenting detailed DVH analysis for future study comparisons. In our study, using S-IMRT for HNSCC patients treated with or without chemotherapy, a mean dose of 68.70Gy and a median dose of 69.50Gy to ≤5% of the ipsilateral BP volume did not result in any BP injury. The mean volume of ipsilateral BP receiving a mean dose of 70Gy (V70) was 1.47 cc, 75Gy (V75) was 0.24 cc suggesting that the volume of BP treated to high dose is relatively small. Even though each axon in the BP functions as a serial structure, the complex, multiple anastomosing arrangements of roots, trunks, divisions, and branches provide a parallel network that could mask and potentially underrepresent symptoms from a focal lesion, while no symptomatic events were identified in this study, the possibility of asymptomatic focal injury could not be excluded.

In treatment planning, attention to the BP will continue to be important to minimize long-term side effects. Chen et al. [2] reported on subjective symptoms experienced by H & N patients related to brachial plexopathy using a symptom questionnaire with a median follow-up of 56 months; in this study 12% of patients reported positive symptoms. Of note 40% of these patients were treated with CRT and 62% of these patients were treated with IMRT using SIB technique where the dose per fraction was as high as 2.12Gy. In our study, with a median follow up of 49 months, all patients were treated using S-IMRT with conventional fractionation and a maximum dose per fraction of 2Gy prescribed to the target volume, with progressively shrinking field resulting in lower dose per fraction and reduced BP volume in the radiation field during treatment of PTV2 and PTV3 and observed no injury to BP in our patients. The differences in dose per fraction and spatial distribution of high dose volume between SIB and S-IMRT technique may have resulted in no injury to BP in our patients. A smaller study of 43 patients from Belgium evaluated H & N cancer patients treated with CRT and found no cases of brachial plexopathy [11]. Radiation therapy in this study was delivered to 35% of patients using IMRT with the remainder of patients treated with 3-dimensional conformal radiotherapy. Of note these patients were treated with 2Gy per fraction daily to 40Gy followed by 1.6Gy twice a day to a total dose of 72Gy in 6 weeks. This suggests that dose per fraction may also play an important role in risk of brachial plexopathy.

The use of CRT may increase the risk of brachial plexopathy although quantifying this risk is difficult. It has been suggested that a range exists from a two-fold effect to higher [12,13]. More recently dose constraints from Amini et al. [14] showed that patients treated for superior sulcus lung cancers with definitive CRT require that a median dose to the BP be kept below 69Gy and the maximum dose to 2 cc below 75Gy to prevent brachial plexopathy. We found no significant difference in the risk of brachial plexopathy for patients treated with or without CRT although most of our patients were treated with CRT.

Over the years dose tolerance as specified from Emami et al. [15] and more recently Quantec [16] note a 5% risk of developing radiation-induced brachial plexopathy at 5 years when one-third, two-thirds, and the whole BP, respectively is treated to 62, 61 and 60 Gy; for a 50% risk at 5 years, the dose tolerances are 77, 76 and 75 Gy respectively. Recent data suggests that dose to the BP in patients undergoing IMRT for head and neck malignancies maybe higher [17]. In our study, with a minimum of 2 years follow-up and mean of 54 months, there were no events of acute or late brachial plexopathy. It may be necessary to give high doses to the BP depending on tumor location; however if lower dose per fraction is given, this may be help to prevent brachial plexopathy although longer follow up is necessary. The limitations of this study include the fact that the evidence of brachial plexopathy was determined by a retrospective review of charts. Furthermore, despite the fact that RTOG guidelines were used to contour the BP and contours were checked by a Head and Neck experienced board certified radiation oncologist, it is extremely difficult to visualize the BP even on a high resolution CT scan and this process is susceptible to over and under contouring. With the evolution of technology, the guidelines for target volume and normal structure definition continue to evolve. Velde et al. [18] recently published anatomically validated contouring guidelines for the BP using cadaver, CT and MRI imaging. The authors concluded that when the BP is outlined using their methodology 100% of the BP was included while only 37.75% of the BP was included when RTOG guidelines were used. This will have a significant impact on dose-volume data. However, this guideline needs further validation by other investigators.

## Conclusion

In this study cohort, at a minimum of two years follow up, the mean dose of 68.7Gy, and a median dose to 69.5Gy to ≤5% of ipsilateral BP did not result in BP injury when patients were treated with S-IMRT technique. However, longer follow up is needed.

## Abbreviations

BP: Brachial Plexus
CRT: Concomitant chemo-radiation
CT: Computed Tomography
CTCAE v3.0: Common Terminology Criteria for Adverse Events version 3.0
CTV: Clinical Tumor Volume
Dmax: Maximum point doses
DVH: Dose Volume Histograms
Dvs: Dose in Gray covering specified percent of brachial plexus volume
ECE: Extra Capsular Extension
GTV: Gross Tumor Volume
HNSCC: Head and Neck Squamous Cell Carcinoma
IMRT: Intensity Modulated Radiation Therapy
IRB: Institutional Review Board
MRI: Magnetic Resonance Imaging
OARs: Organs at risk
PTV: Planning Tumor Volume
ROI: Region of interest
RTOG: Radiation Therapy Oncology Group
SIB: Simultaneous Integrated Boost
S-IMRT: Sequential Intensity Modulated Radiation Therapy
Vds: Brachial Plexus volumes receiving a specified dose
